# Entrapment of *Citrus* *limon* var. *pompia* Essential Oil or Pure Citral in Liposomes Tailored as Mouthwash for the Treatment of Oral Cavity Diseases

**DOI:** 10.3390/ph13090216

**Published:** 2020-08-28

**Authors:** Lucia Palmas, Matteo Aroffu, Giacomo L. Petretto, Elvira Escribano-Ferrer, Octavio Díez-Sales, Iris Usach, José-Esteban Peris, Francesca Marongiu, Mansureh Ghavam, Sara Fais, Germano Orrù, Rita Abi Rached, Maria Letizia Manca, Maria Manconi

**Affiliations:** 1 Department of Scienze della Vita e dell’Ambiente, Drug Science Division, University of Cagliari, 09124 Cagliari, Italy; luciapalmas@yahoo.it (L.P.); matteo.aroffu@gmail.com (M.A.); f.marongiu@unica.it (F.M.); manconi@unica.it (M.M.); 2 Department of Chemistry and Pharmacy, University of Sassari, 07100 Sassari, Italy; gpetretto@uniss.it (G.L.P.); jose.e.peris@uv.es (J.-E.P.); 3 Biopharmaceutics and Pharmacokinetics Unit, Institute for Nanoscience and Nanotechnology, University of Barcelona, 08193 Barcelona, Spain; eescribano@ub.edu; 4 Department of Pharmacy, Pharmaceutical Technology and Parasitology, University of Valencia, Burjassot, 46100 Valencia, Spain; octavio.diez@uv.es (O.D.-S.); Iris.Usach@uv.es (I.U.); 5 Department of Range and Watershed Management, Faculty of Natural Resources and Earth Sciences, University of Kashan, Kashan 8731753153, Iran; mansurehghavam@gmail.com; 6 Department of Surgical Science, University of Cagliari, Molecular Biology Service Lab (MBS), Via Ospedale 40, 09124 Cagliari, Italy; sara.fais@unica.it (S.F.); orru@unica.it (G.O.); 7 Centre d’Analyses et de Recherche, Unité de Recherche TVA, Laboratoire CTA, Faculté des Sciences, Université Saint-Joseph, B.P. 17-5208 Riad El Solh, Beirut 1104 2020, Lebanon; rita.abirached@net.usj.edu.lb

**Keywords:** *Citrus limon* essential oil, hydrogen peroxide, keratinocyte migration, scratch assay, anticaries activity, *Streptococcus mutans*

## Abstract

This work aimed at developing a mouthwash based on liposomes loading *Citrus limon* var. *pompia* essential oil or citral to treat oropharyngeal diseases. Vesicles were prepared by dispersing phosphatidylcholine and pompia essential oil or citral at increasing amounts (12, 25 and 50 mg/mL) in water. Transparent vesicle dispersions were obtained by direct sonication avoiding the use of organic solvents. Cryogenic transmission electron microscopy (cryo-TEM) confirmed the formation of unilamellar, spherical and regularly shaped vesicles. Essential oil and citral loaded liposomes were small in size (~110 and ~100 nm, respectively) and negatively charged. Liposomes, especially those loading citral, were highly stable as their physico-chemical properties did not change during storage. The formulations were highly biocompatible against keratinocytes, were able to counteract the damages induced in cells by using hydrogen peroxide, and able to increase the rate of skin repair. In addition, liposomes loading citral at higher concentrations inhibited the proliferation of cariogenic bacterium.

## 1. Introduction

Since ancient times, human beings have resorted to natural remedies to either heal wounds or treat disease [1] and plants have always been a primary source of active compounds. Among them, essential oils are of remarkable interest. They are plant-derived blends (phytocomplexes) composed of lipophilic and highly volatile secondary metabolites, basically synthesized by every organ of the plant [2,3].

Nowadays, some of them, including those obtained from Citrus (i.e., *Citrus bergamia, Citrus limon, Citrus aurantifolia, Citrus aurantium, Citrus reticulate, Citrus sinensis*), are reported on the GRAS list and are generally recognised as safe by the FDA [4]. They have been used for many years in both the medical and cosmetic fields, as well as for alimentary and domestic purposes. The first written record about their use in the medical field are related to the *Cedrus* spp. oil and have been reported in ancient Mesopotamian texts [5]. Since then, their use and interest has been increasing throughout the centuries due to the wide variety of beneficial activities they can exert. This activity is clearly related to both type and composition of the essential oil. At first, they were used due to their antimicrobial properties. A recent review discloses that the essential oils of lavender, thyme, peppermint, cajuput, cinnamon, clove, sage, eucalyptus and tea tree are active and effective against viruses such as Herpes simplex virus or influenza virus H1N1 and against clinically relevant pathogens such as *Staphylococcus aureus*, *Escherichia coli*, *Pseudomonas aeruginosa* among others, and even against resistant strains [6]. Proven antimicrobial activity is also ascribed to the *Citrus* spp. essential oils [7]. Among them, the *Citrus limon* var. *pompia* essential oil has been tested to prevent the growth of polymicrobial biofilms composed of *Pseudomonas aeruginosa* and pathogenic fungi such as *Candida albicans* [8]. Likewise, in the food sector it has been used to evaluate the inhibition of the activity of food-related pathogens such as *Listeria monocytogenes* and *Staphylococcus aureus* [9]. In addition, *Citrus* spp. essential oils are also well known due to their antioxidant properties [10,11], like those of oregano and thyme too and can be used to prevent the oxidation of greasy food [12].

In the medical field the antioxidant activity of essential oils has instead been evaluated to counteract the proliferation activity of tumoral cells in either prostate, breast or lung cancers [13], and some of them could even be used as a supportive therapy in oncology [14]. Moreover, the use of essential oils in mind-related disorders such as Alzheimer’s disease [15], anxiety disorders [16] and postoperative nausea and vomiting [17] is currently under assessment.

Bearing in mind all these activities associated with the use of essential oils, it goes without saying that the pharmaceutical, cosmetics and food industries are particularly interested in research of methods or systems able to improve the activities of the bioactives contained in the essential oils. Their loading in nanocarriers has been shown as an approach to bet on [2]. Indeed, it may limit problems such as low water solubility and high volatility along with degradation of essential oils by environmental factors, usually improving their bioavailability to the target tissue and therefore in vivo activity [18]. Previous studies have shown the optimal performance achieved by liposomes in the delivery of natural active ingredients [19] as well as essential oils [20]. Additionally, because of the peculiar lamellar structure and amphipathic nature of their main components (phospholipids), they seem to be especially suitable for the loading of lipophilic components of essential oil inside the bilayer of vesicles, which are stably dispersed in an aqueous phase miscible with biological fluids [21]. Indeed, different essential oils have been loaded in liposomes resulting in alternative therapeutic agents to treat several diseases [22]. The essential oil of *Artemisia arborescens* L. was successfully loaded in liposomes providing a better antiviral activity in comparison with the free essential oil [23]. Similar outcomes were achieved delivering *Santolina insularis* essential oil within liposomes [24]. 

The essential oil of *Thymus capitatus,* especially when loaded in ad hoc formulated phospholipid vesicles, exerted antioxidant, regenerative and antibacterial activities in epithelial tissues [25]. The liposome-loaded essential oil of *Citrus limon* var. *pompia* disclosed antibacterial properties against *E. coli*, *S. aureus* and *C. albicans* [20]. Due to their activity both of these encapsulated-oils can also prevent dental decay [26]. Therefore, the obtained results underlined the key role played by nanocarriers in improving the effectiveness of essential oils. 

Taking into account these promising findings, the essential oil obtained from *Citrus limon* var. *pompia* was loaded into liposomes by using increasing amounts of it up to the critical concentration, which caused an excessive increase of vesicle size. Alternatively, citral was loaded into liposomes in the same amount, as it is one of the most abundant terpenes contained in *Citrus* spp. essential oils, widely used as a flavour component in the food, beverage and fragrance industries due to its pleasant aroma [20,27]. It is able to inhibit the proliferation of several pathogens, especially in food [28].

Liposomes were characterized for their structure and morphology by cryogenic-transmission electron microscopy (Cryo-TEM) and for their mean diameter and zeta potential by means of dynamic light scattering. The biocompatibility of liposomes along with their ability to protect cells from the damages caused by oxidative stress and to promote the closure of lesions induced in a cell monolayer has been evaluated by using keratinocytes. Moreover, the inhibitory activity of the essential oil or citral loaded in the vesicles against *Streptococcus mutans (S. mutans)* was evaluated.

## 2. Results

### 2.1. Vesicle Characterization

**Figure 1:**
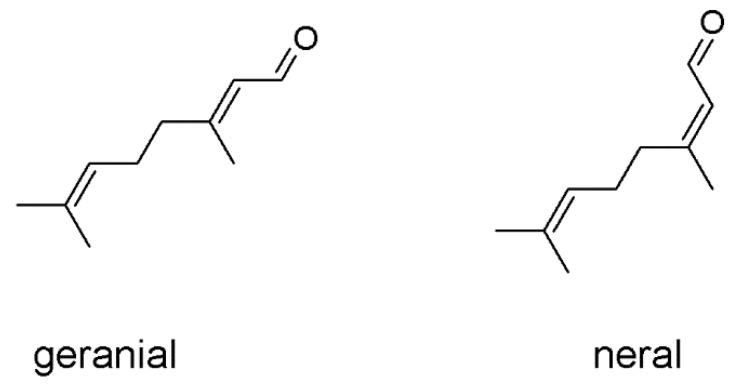
Chemical structure of both geranial and neral, which form the citral mixture.

The essential oil of *Citrus limon* var. *pompia* was previously characterized and it displayed promising antibacterial properties against *E. coli*, *S. aureus* and *C. albicans* [20]. Its main component is citral, a mixture of two geometric isomers of an aldehyde monoterpene: geranial is the Z-isomer and neral is the E-isomer (Figure 1). Liposomes have been previously used to deliver several natural molecules, extracts or essential oils, demonstrating them to be ideal carriers capable of improving the efficacy of the incorporated bioactives, especially in the skin and mucosae [20]. Taking into account these studies, the *pompia* essential oil was loaded in liposomes at increasing concentrations (12, 25, and 50 mg/mL). Considering that citral is one of the main components of the *pompia* essential oil and its antimicrobial properties are well known, citral loaded liposomes were formulated as well, by using the same concentrations (12, 25, and 50 mg/mL). Empty liposomes were also prepared and used as reference, aiming at evaluating the effect of *pompia* essential oil or citral on the vesicle assembling.

**Figure 2:**
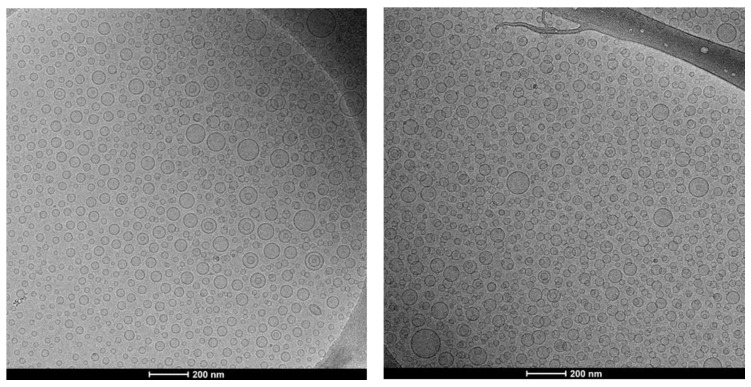
Representative cryo-TEM images of essential oil (50 mg/mL) loaded liposomes (**left panel**), and citral (50 mg/mL) loaded liposomes (**right panel**).

Morphology and structure were observed by cryo-TEM. Micrographs showed that all the vesicles were mainly spherical and unilamellar indicating that the loading of essential oil or citral did not interfere within the organization of both the structure of vesicles and assembling of the phospholipid bilayer (Figure 2).

The mean diameter of the vesicles was measured by dynamic laser light scattering, which provided a global estimation of the vesicle size calculated from the scattering intensity of each particle fraction (Table 1).

The empty liposomes showed a mean diameter around 130 nm but the incorporation of essential oil or citral led to a reduction in the average size, indeed, oil loaded liposomes were smaller (~114 nm, *p* < 0.05 versus empty vesicles) no matter the amount of oil used. Liposomes loading 25 and 50 mg/mL of citral were smaller than the corresponding vesicles loading essential oil, indeed, their mean diameter was ~98 nm (*p* < 0.05 versus essential oil loaded liposomes). The mean diameter of liposomes loading 12 mg/mL of citral was 105 nm, equal (*p* > 0.05) to that of 12 mg/mL of essential oil and equal (*p* > 0.05) to that of liposomes loading 25 and 50 mg/mL of citral.

All samples were polydisperse (polydispersity index between 0.268 and 0.387) indicating the presence of more than one dimensional population. Nevertheless, the results of size and polydispersity index were highly reproducible as shown by the small standard deviations values. The values of zeta potential were highly negative for all the formulations, especially for essential oil-loaded liposomes (~−85 mV) compared to citral-loaded vesicles (~−72 mV). The entrapment efficiency was around 89%, confirming that the amount of essential oil or citral incorporated in the vesicles was very high.

**Figure 3:**
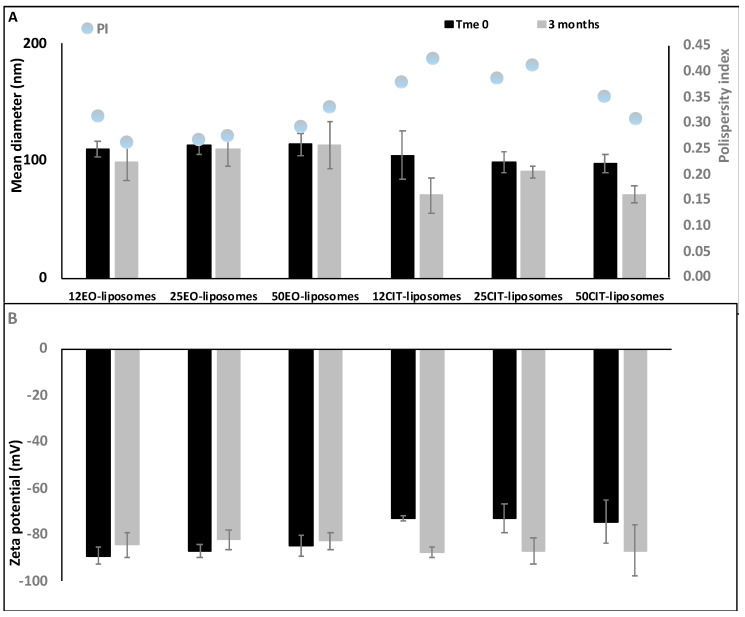
Mean diameter, polydispersity index (**A**) and zeta potential (**B**) of *pompia* essential oil or citral containing liposomes during 90 days of storage. Mean values ± standard deviation of at least three replicates are reported.

The stability of liposomes was evaluated for 90 days, storing the samples at 25 °C and measuring their physico-chemical properties by DLS every 30 days. (Figure 3).

The results highlighted that all the formulations were highly stable since only a small variation of the size was pointed out (±10%) during the study. Size of citral loaded vesicles slightly decreased while their value of zeta potential became more negative although not significantly.

### 2.2. Biocompatibility of Vesicles and Protective Activity against Oxidative Stress

**Figure 4:**
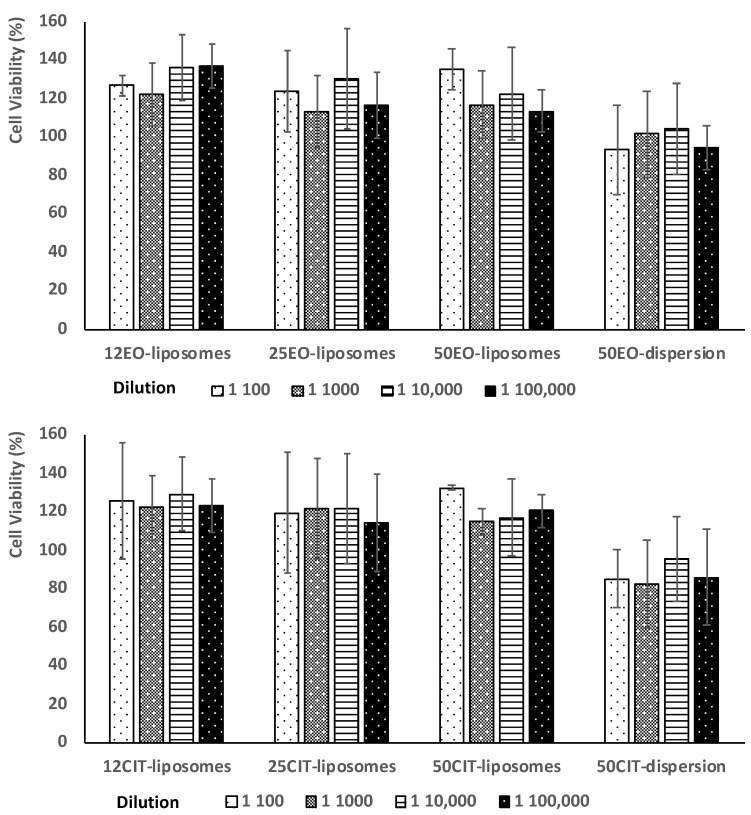
Viability of keratinocytes incubated for 48 h with *pompia* essential oil (**upper panel**) or citral (**lower panel**) by using four different dilutions. Data are reported as mean values ± standard deviation of cell viability expressed as the percentage of untreated cells (100% of viability).

To evaluate biocompatibility and antioxidant activity of the formulations, human keratinocytes were used as they represent 90% of the epithelial cells in the oral mucosa [29]. The biocompatibility of the formulations was evaluated by incubating keratinocytes with liposomes at four different concentrations of essential oil or citral for 48 h. Dispersions of essential oil or citral in water and Tween 80 were prepared and used as references (Figure 4).

Results demonstrate no toxic activity against epithelial cells. Indeed, the viability of cells treated with essential oil or citral in dispersions was around 100% regardless of the dilution tested (*p* > 0.05 among the different dilution of the dispersion). The loading of the oil or citral in liposomes slightly increased cell viability up to ~112%, without statistically significant difference among the used payloads, concentrations and dilutions (*p* > 0.05). 

**Figure 5:**
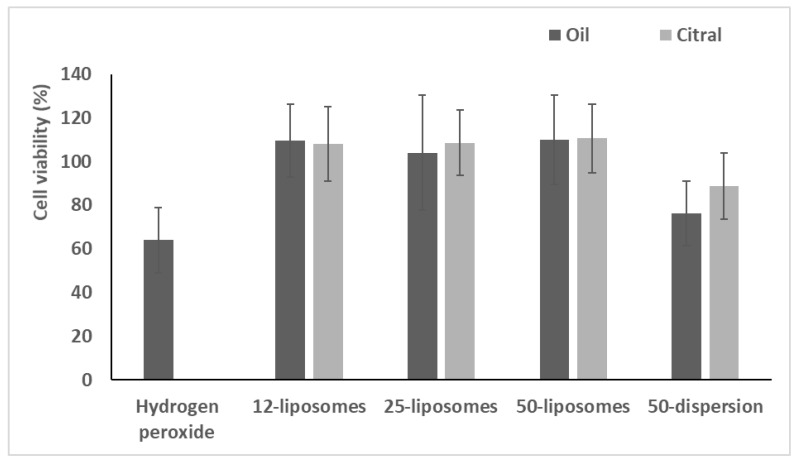
Viability of cells treated with essential oil or citral in dispersions or loaded in liposomes and stressed with hydrogen peroxide. Data (bars) are reported as mean values ± standard deviations of cell viability expressed as the percentage of untreated cells (100% viability).

The protective effect of the formulations incorporating essential oil or citral against stressed keratinocytes was evaluated, and dispersions of essential oil or citral in water and Tween 80 were prepared and used as references (Figure 5).

After exposure for 4 h with hydrogen peroxide, the viability of stressed cells was ~60%. The use of oil or citral in dispersions did not effectively reduce the damage induced by hydrogen peroxide producing a viability ~80%. (*p* > 0.05 versus cells stressed with hydrogen peroxide and untreated). The treatment with essential oil or citral loaded vesicles significantly counteracted the damages induced by oxidative stress, restoring the viability up to ~110% (*p* < 0.05 versus stressed and untreated cells). No significant difference was detected between *pompia* essential oil and citral loaded liposomes, *p* > 0.05.

These results indicated that essential oil and citral loaded liposomes avoided the toxic effects connected with oxidative stress mainly because of the antioxidant properties of the bioactives [8,11].

### 2.3. Improvement of Proliferation and Migration of Keratinocytes

**Figure 6:**
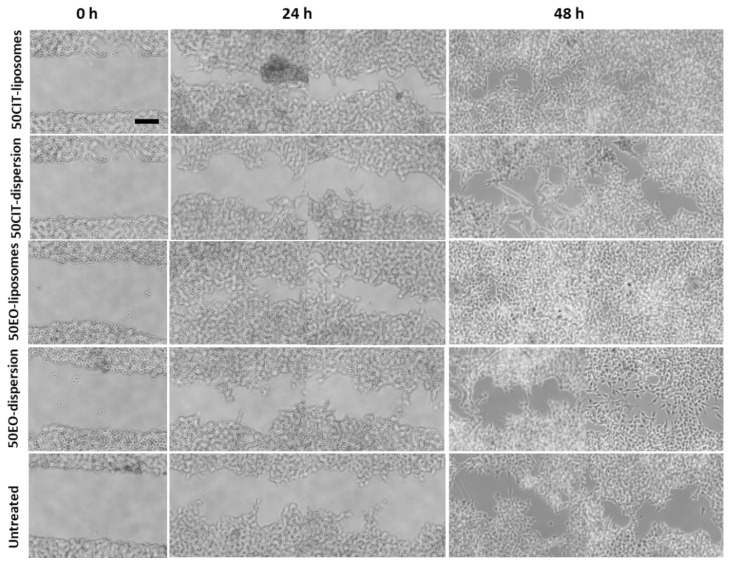
Representative optical microscopy images of wound closure in keratinocytes followed by the treatment with essential oil or citral in dispersion or loaded in liposomes for 0, 24 and 48 h. Bar corresponds to 200 µm.

The ability of the formulations to promote both proliferation and migration of keratinocytes was assessed by means of the scratch assay, which involves the evaluation of cells spreading on the wound area, consisting of a linear cut into a cell monolayer (Figure 6).

The scratch in untreated keratinocytes was basically unchanged after 24 h and slightly increased at 48 h. Contrastingly, the lesion was tighter in cells treated with *pompia* essential oil or citral in dispersion.

**Figure 7:**
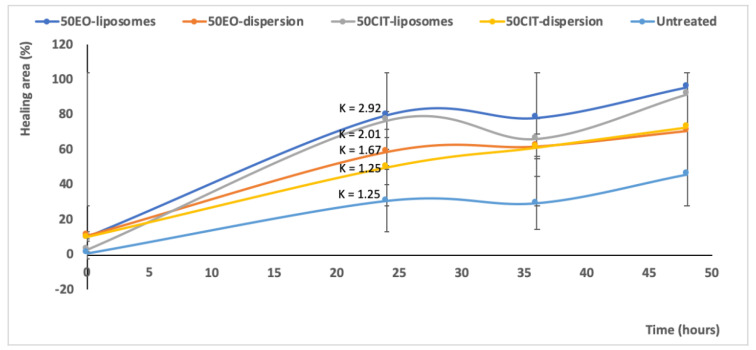
Values of healing area of the scratch induced in a monolayer of keratinocytes treated with *pompia* essential oil or citral in dispersion or loaded in liposomes. Measures were performed at different time points (0, 24, 36, 48 h) and the K indicated the slope of each curve in the first tract. Mean values ± standard deviations are reported.

The treatment with essential oil or citral loaded liposomes stimulated cell proliferation and migration to a greater extent in comparison with the corresponding dispersions, providing a significant reduction of the width of the wound and an almost complete closure of the scratch. Indeed, after 48 h, the wound was almost closed in all the cells treated with the essential oil loaded liposomes (96% healing) and citral loaded liposomes (92% healing) irrespective of the concentration of bioactive compound used. For the sake of clarity, only the images of plates treated with the highest concentration of essential oil or citral loaded vesicles have been reported. The wounded areas from different pictures were also measured, the values were reported at different times and the closure rate was calculated from the slope of the linear regression (Figure 7). The closure of untreated cells was constant during the experiment and at 48 h was not completed (~50%). The wound closure of the cells treated with the oil or citral in dispersion or loaded in vesicles was very rapid during the first 24 h and thereafter increased slowly. Therefore, the mean rate (K) of closure was measured in the first portion of the curve. The rate of the untreated cells and citral in dispersion was the lowest (~1.25 mm^2^/h), that of essential oil in dispersion was slightly higher (~1.67 mm^2^/h) and that of bioactives loaded in liposomes was the highest, especially that of essential oil loaded liposomes (~2.92 mm^2^/h). This result indicated that the essential oil was more effective in tissue regeneration especially when loaded into liposomes. 

### 2.4. Inhibitory Activity of Formulations against S. mutans

**Figure 8:**
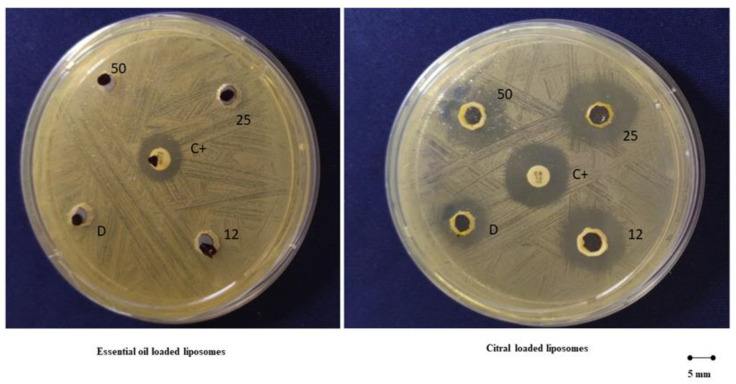
Antimicrobial diffusion method of 12, 25 and 50 mg/mL of essential oil in dispersion or loaded in liposomes (left panel) and 12, 25 and 50 mg/mL of citral in dispersion or loaded in liposomes (right panel). Gentamicin (10 mg/disk) represents the positive control (C+).

The antibacterial activity of liposomes loading the essential oil or the citral at the highest concentration (50 mg/mL) was evaluated by measuring the diameter of the inhibition halo, a clear zone of bacterial growth inhibition. This preliminary result demonstrated that essential oil loaded liposomes did not inhibit *S. mutans* proliferation (halo inhibition <5 ± 1 mm Ø) while citral loaded liposomes provided an inhibition halo of 16 ± 1 mm Ø, whereas citral in dispersion delivered a lower inhibition diameter (11 ± 1 mm Ø) (Figure 8).

## 3. Discussion

The dispersions of liposomes loading *pompia* essential oil or citral appeared to be ideal candidates for the treatment of oral cavity diseases. Indeed, the resulted dispersions were yellow-transparent, maintained surface brightness and had a good visual appearance probably due to the high content of citral, which also ensured an intense and pleasant flavour. Citral (3,7-dimethyl-2,6-octadienal) is a blend of two geometric isomers called geranial and neral, which are monoterpene aldehydes [30]. It is well known for its fresh citrus aroma and taste [31]. Vesicles were small in size thanks to the suitable intercalation of citral and other terpenes inside the bilayer (~110 nm). Indeed, the empty liposomes prepared without oil or citral were bigger, in line with previous results that highlighted the small decrease in size of the vesicles after loading essential oils or lipophilic drugs as a result of an higher cohesion among the phospholipids [23,32]. Vesicle dispersions were stable during storage and biocompatible with epithelial cells, confirming their optimal suitability for treatment of oral cavity diseases. They were demonstrated to be promising antioxidant agents able to protect cells, which in this area are regularly subjected to oxidative stress. In fact, being the main access for food, the oral cavities are continuously exposed to many external chemical agents or mechanical factors, which cause alterations in the equilibrium of the endogenous microbiota formed by commensal and pathogenic microorganisms that live in symbiotic conditions forming a biofilm based on mutual benefit [33,34]. The ability of *pompia* essential oil to counteract oxidative stress has not been previously demonstrated and can be related to different terpenes contained in the essential oils. In fact, they are able to react with peroxyl radicals, as demonstrated by *Origanum vulgaris* essential oil, whose most relevant compounds are thymol and carvacrol [9,12], *Thimus capitatus* essential oil, whose most relevant compound is still carvacrol [25], and many others [35,36]. A comparable antioxidant activity was also exerted by the citral loaded liposomes due to citral, an aldehydic terpene [37], as previously reported [38,39,40].

Additionally, the tested formulations disclosed promising properties also in the scratch assay, demonstrating the almost complete closure of the wound after 48 h of treatment. This important finding is in accordance with a previous study, which reported that a mouthwash containing essential oils displayed no detectable detrimental effects on human gingival and fibroblasts whereas the one containing chlorhexidine reduced both cell migration and cell long-term survival [41]. 

Among the prepared formulations, bearing in mind the aim is to obtain a mouthwash for the treatment of oral caries, liposomes loading citral (50 mg/mL) appeared to be more appealing thanks to their inhibitory effect versus *S. mutans*. This one is a microorganism able to acquire new properties leading to the expression of pathogenicity determinants which cause its virulence in specific environmental conditions. Among them, a local decrease of pH arising from the production of weak organic acids by the metabolism of bacteria, can result in the demineralization of teeth surfaces allowing *S. mutans* to adhere to them generating dental plaque. As is well known, if it persists for long periods this can induce a cavitation where microorganisms aim to adapt themselves fuelling this process [42]. Moreover, external factors like cigarette smoke, food, alcohol and oral bacteria, can determine the production of ROS and trigger an inflammatory response as a defence against the organisms causing oxidative stress. The excessive accumulation of free radicals is an additional factor which promotes the disruption of the equilibrium of the oral biofilm facilitating, as a consequence, the proliferation of pathogens from the oral cavity such as *S. mutans*, one of the many etiological factors of dental caries [42,43]. Considering this important effect exerted by citral loaded liposomes, this formulation possesses all the requirements to be considered an effective, safe and pleasant mouthwash for the treatment of oral cavity diseases.

## 4. Materials and Methods

### 4.1. Materials

*Pompia* essential oil was extracted from *pompia* leaves collected in 2017 in Siniscola (Sardinia, Italy). Of these leaves, 250 g were suspended in 700 mL of water and subjected to steam distillation using a Clevenger type apparatus as previous reported [20]. Lipoid S75 (S75), a mixture of soybean phospholipids (70% phosphatidylcholine, 9% phosphatidyletanolamine and 3% lysophosphatidylcholine), triglycerides and fatty acids, was purchased from Lipoid GmbH (Ludwigshafen, Germany). Methanol, citral and all other reagents were purchased from Sigma-Aldrich (Milan, Italy). Cell medium, foetal bovine serum, penicillin and streptomycin were purchased from Life Technologies Europe (Monza, Italy).

### 4.2. Liposome Preparation

*Pompia* essential oil or citral (12, 25 and 50 mg/mL) and S75 (60 mg/mL) were weighted in a glass vial, mixed together and then dispersed in water. The dispersions were left hydrating for 24 h at 25 °C to facilitate the swelling of the phospholipid (S75), and then sonicated (5 s on and 2 s off, 20 cycles; 13 microns of probe amplitude) with a high intensity ultrasonic disintegrator (Soniprep 150, MSE Crowley, London, UK). Empty liposomes without essential oil or citral were prepared as well and used as reference. The vesicle dispersions (1 mL) were purified from the non-entrapped essential oil or citral by dialysis method by using 3 L of water (by using Spectra/Por membranes (12–14 kDa MW cut-off, 3 nm pore size; Spectrum Laboratories Inc., DG Breda, The Netherlands). The dialysis was carried out for 2 h at 25 °C under constant stirring, refreshing the medium after 1 h. The concentration of the main components of essential oil or citral was measured in non-dialyzed and dialyzed liposomes. The entrapment efficiency was calculated as the percentage of the amount of active components recovered in the dialysed liposomes versus that found in non-dialysed liposomes. The concentration of components was measured by gas chromatography as previously reported [20]. Before the analysis, liposomes were disrupted by diluting them in methanol. 

### 4.3. Liposome Characterization

Liposome formation and morphology were evaluated by cryogenic transmission electron microscopy (cryo-TEM). Each sample was adsorbed on a grid covered with a Holey carbon film and transferred in an automatic plunge freezing apparatus (Vitrobot, FEI, Eindhoven, The Netherlands) to control humidity and temperature. The resulting film was vitrified by plunging the grid (kept at 100% humidity and room temperature) into ethane, maintained at its melting point, using a Vitrobot (FEI Company, Eindhoven, The Netherlands) and then observed at −173 °C in a Tecnai F20 microscope (FEI Company) operating at 200 kV. Digital images were acquired using low-dose imaging conditions with a CCD Eagle camera (FEI Company).

The average diameter and polydispersity index (a measure of the width of size distribution) of vesicles were determined by dynamic light scattering by using a Zetasizer Ultra (Malvern Panalytical, Worcestershire, UK). The zeta potential was estimated by using the Zetasizer Ultra, which converts the electrophoretic mobility by means of the Smoluchowski approximation of the Henry equation. Samples (*n* = 6) were diluted (1:100) with water, and analyzed at 25 °C.

### 4.4. Stability Study

The stability of the formulations were evaluated by keeping them in the dark at 25 °C for 3 months and by measuring vesicle average size, polydispersity index and zeta potential each month.

### 4.5. Cell Viability and Protection against Oxidative Stress

Immortalized human keratinocytes (HaCaT) were grown as monolayers at 37 °C, 100% humidity and 5% CO_2_, using Dulbecco’s Modified Eagle Medium (DMEM) with high glucose, supplemented with 10% (*v*/*v*) foetal bovine serum, penicillin (100 U/mL), and streptomycin (100 µg/mL) as growth medium. The cells were seeded into 96-well plates at a density of 7.5 × 10^3^ cells/well and incubated for 24 h. Then, cells were treated for 48 h with different concentrations of essential oils or citral loaded liposomes properly diluted with DMEM (1:100, 1:1000, 1:10,000, 1:100,000). The essential oil or citral dispersed in an aqueous solution containing Tween 80 (1%) was used at the same concentrations, for comparison. Cell viability was determined by adding MTT (3(4,5-dimethylthiazolyl-2)-2,5-diphenyltetrazolium bromide) (100 µL, 0.5 mg/mL final concentration) to each well, dissolving (after 3 h) the formed formazan crystals with DMSO, and measuring the absorbance at 570 nm by using a microplate reader (Synergy 4 Reader, BioTek Instruments, AHSI S.p.A, Bernareggio, Italy). All experiments were repeated at least three times, each time in triplicate. Results are shown as percent of cell viability in comparison with non-treated control cells (100% viability).

The in vitro protective effect of the liposomes against cell damages caused by oxidative stress was evaluated. The cells were seeded into 96-well plates at a density of 7.5 × 10^3^ cells/well and incubated for 24 h. Subsequently, cells were stressed with hydrogen peroxide (1:40,000 dilution) and treated with the essential oil or citral loaded liposomes properly diluted with DMEM (1:10,000). For comparison, cells were also treated with essential oil or citral dispersed in an aqueous solution containing Tween 80 (1%) at the same dilution. Cells treated only with hydrogen peroxide were used as a positive control. After 4 h of incubation, the cells were washed with fresh medium, and their viability was determined by the MTT assay as described above. The results are reported as the percentage of untreated cells (100% viability).

### 4.6. In Vitro Scratch Assay

The ability of liposomes containing essential oil or citral to stimulate HaCaT proliferation and migration was evaluated by measuring the cell expansion on the wound surface (scratch assay). The cells were cultured in 6-well plates and, when the complete confluence was reached, a linear scratch was generated. The scattered cells were removed by gently washing with fresh medium. The cells were treated with *pompia* essential oil or citral in dispersion or loaded in liposomes (dilution 1:10,000) and incubated for 48 h [44]. Untreated cells were used as control. The speed of cell migration and consequent wound closure was observed under a light microscope using a 10× objective, thus assessing the efficacy of the formulations on closure of skin lesions. The lesion areas have been measured from different images, the percentage of the healing areas was calculated with respect to the lesion values of untreated cells. The values were reported in a graph as a function of the time and the healing rate was calculated from the slope of the linear regression.

### 4.7. Antimicrobial Activity

The oral pathogen *S. mutans* CIP103220 (Collection Institut Pasteur), was used as target- bacterium to evaluate the antimicrobial activity of the studied formulations. Antimicrobial activity of *pompia* essential oil, or citral loaded vesicles, was assessed using the highest concentration of the payloads and a susceptibility test was performed by using a modified agar diffusion test, already described by Orrù et al. [29]. Following this, 90 mm Ø Petri dishes containing 15 mL of Shaedler agar (Microbiol, Uta Cagliari) were inoculated with an *S. mutans* 1 × 10^7^ CFU/mL suspension and 15 µL of each formulation was placed in a well (Ø 5 mm) located in the centre of the plate. The dishes were incubated in air at 37 °C and 5% CO_2_ for 24 h species, the diameter of the possible inhibition alone was measured, and the experiment was performed in triplicate. On the other hand, gentamicin (10 mg/disc, Gibco, Monza MB, Italy) was used as a positive control. The biological tests were conducted in a Class II type A2 biological safety cabinet, in accordance with the protocols of the Clinical and Laboratory Standards Institute (CLSI).

### 4.8. Statistical Analysis of Data

The results were expressed as mean values ± standard deviation. Statistically significant differences among samples were determined using variance analysis. The ad hoc post Tukey–Kramer *t*-test was used to substantiate a significant difference between the means of two specific groups. The statistical analysis was performed by using the Excel software package (Microsoft Corp, Redmond, WA, USA) equipped with a tool for statistical analysis. The minimum level of significance chosen was *p* < 0.05.

## 5. Conclusions

The results of this comparative study underline that the incorporation of essential oil or citral in phospholipid vesicles provides the formation of yellow-transparent dispersions having a good visual appearance and an intense and pleasant flavour, thus they seem to be suitable to formulate a mouthwash. The vesicle loading also enhances the efficacy of the payloads improving the protection against oxidative stress and accelerating the healing of wounded mucosa. Liposomes loading 50 mg/mL of citral appeared as the most promising dispersion since they were also able to inhibit the proliferation of *S. mutans*. Thus, this formulation may represent the starting point to formulate an effective, safe and pleasant mouthwash to control oral hygiene and health. By the way, its proven regenerative and protective efficacy should be enhanced by adding a natural antioxidant.

## Figures and Tables

**Table 1:** Mean diameter (MD), polydispersity index (PI), zeta potential (ZP) and entrapment efficiency of *pompia* essential oil (EO) or citral (CIT) loaded liposomes. Mean values ± standard deviation of at least three replicates are reported. Each symbol *, ^#^, ^+^ and ^§^, indicates the same value.

Formulation	MD (nm)	PI	ZP (mV)	EE (%)
Empty liposomes	130 ± 9 *	0.430 ± 0.097	−60 ± 8	-
12EO-liposomes	110 ± 7 ^#,+^	0.312 ± 0.094	−89 ± 4	86 ± 11
25EO-liposomes	114 ± 8 ^#^	0.268 ± 0.039	−87 ± 3	91 ± 8
50EO-liposomes	117 ± 9 ^#^	0.291 ± 0.084	−85 ± 5	87 ± 10
12CIT-liposomes	105 ± 8 ^§,+^	0.377 ± 0.020	−73 ± 1	94 ± 13
25CIT-liposomes	99 ± 9 ^§^	0.387 ± 0.032	−70 ± 8	91 ± 11
50CIT-liposomes	97 ± 7 ^§^	0.351 ± 0.083	−74 ± 9	89 ± 10
